# The hamster wheel: a case study on embodied narrative identity and overcoming severe obesity

**DOI:** 10.1007/s11019-021-10002-x

**Published:** 2021-01-13

**Authors:** Eli Natvik, Målfrid Råheim, Randi Sviland

**Affiliations:** 1 Department of Health and Caring Sciences, Western Norway University of Applied Sciences, Førde, Norway; 2 The Centre for Health Research, District General Hospital of Førde, Førde, Norway; 3 Department of Global Public Health and Primary Care, University of Bergen, Bergen, Norway; 4 Department of Health and Functioning, Western Norway University of Applied Sciences, Bergen, Norway

**Keywords:** Weight loss surgery, Bariatric care, Lived body, Narrative, Obesity/overweight, Phenomenology, Selfhood, Weight loss maintenance, Weight cycling, Ricoeur, Merleau-Ponty

## Abstract

Based in narrative phenomenology, this article describes an example of how lived time, self and bodily engagement with the social world intertwine, and how our sense of self develops. We explore this through the life story of a woman who lost weight through surgery in the 1970 s and has fought against her own body, food and eating ever since. Our narrative analysis of interviews, reflective notes and email correspondence disentangled two storylines illuminating paradoxes within this long-term weight loss process. *Thea’s Medical Weight Narrative: From Severely Obese Child to Healthy Adult* is her story in context of medicine and obesity treatment and expresses success and control. *Thea’s Story: The Narrative of Fighting Weight* is the experiential story, including concrete examples and quotes, highlighting bodily struggles and the inescapable ambiguity of being and having one’s body. The two storylines coexist and illuminate paradoxes within the weight loss surgery narrative, connected to meaningful life events and experiences, eating practices and relationships with important others. Surgery was experienced as lifesaving, yet the surgical transformation did not suffice, because it did not influence appetite or, desire for food in the long run. In the medical narrative of transforming the body by repair, a problematic relationship with food did not fit into the plot.

## Introduction

“I had weight loss surgery in 1975, and I never hear anything about how the people who had surgery in the 70ties and 80ties are doing. Today, I am nearly normal weight, but when I had surgery, I was 140 kg. It has been a long journey to get to where I am today. You are welcome to hear my story.”

This e-mail was a response to previous research findings and sparked the idea of writing a narrative case study with a life course perspective. Thea was in her early sixties. Forty-five years after weight loss surgery (WLS) she shared her life story revolving around body weight. Her story is analysed in light of phenomenological and narrative theory, and cultural narratives surrounding current WLS stories. Starting from narrative phenomenology, we seek to understand how experience of the world, oneself and others are told, formed, and influenced by the body. In the medical narrative genre of WLS kilos and Body Mass Index, are core concepts. Life is lived, however, and is told from the first-person perspective, and always situated within a cultural context of time and place, and narratives shaped by the medical culture tend to weave into the individual person’s particular narrative.

With Thea’s narrative we aim to investigate the complexity of dealing with being overweight over time. To understand core meanings of selfhood, body and change, we explore her rich narrative account of struggling with her body weight, feelings and eating for a lifetime. We investigate interconnections between a life story, body and self through the lens of weight loss and weight loss maintenance following surgery. Our inquiry departed from the following questions: What is it like to keep fighting for a healthy weight throughout life? In which ways do fighting weight weave into self-understanding and relationships? How does Thea’s life story and weight story interweave and make meaning?

First, we lay out the theoretical lens of this analysis combining a phenomenological and narrative perspective on embodied life. We then present the methodological underpinning before sketching out the method of producing this narrative material. Thea’s narrative is structured into two distinct genres: a brief medical narrative and a life story. Finally, in the discussion we aim to deepen the understanding of her struggles with food, body and emotions throughout life and in the wake of surgically induced weight loss.

### Embodied narrative phenomenology: a theoretical lens

In phenomenology, the *first-person perspective* is decisive*,* meaning that any understanding of experience begins from the subjective dimension. Merleau-Ponty described the body as foundational for human existence, a premise for having experiences, thoughts and emotions, a past, a present and an anticipated future (Merleau-Ponty 1945/2012; Landes 2017). We experience ourselves related to others and through others, in action and interaction (Merleau-Ponty 1945/2012). In other words, subjects depend on and inhabit an *intersubjective* world from the very start. Human existence is always already embodied and situated in both nature and culture*.* The notion of the *lived body* points to the subjective (thoughts and emotions) and physical dimensions of our being as inseparable, and at the same time our reaching toward and inhabiting of the sociocultural world. The body is both me and mine, I am it and I have it, I can see from it, I am visible as it. I cannot get rid of it and it will outlive me in the sense that as matter it lasts longer than I. As such, understanding the body as lived, recognizes a certain ambiguity (Merleau-Ponty 1945/2012).

Emphasizing subjectivity and the lived body does not mean that individuality takes precedence over the world of physical things and others, or that a specific truth resides deep inside each person (Merleau-Ponty 1945/2012). Rather than being encapsulated within itself, the individual exists primarily through preconscious, pre-reflective dealing with the physical and sociocultural world (Slatman 2014). The phenomenological foregrounding of the lived body and the inseparability between person and world makes it possible to bypass traditional distinctions between body and soul, nature and culture, flesh, thought and emotions, person and world. We have our bodies, are our bodies, and we are more.

Slatman (2014) pointed out that our *own* (lived) body always carry *strange* elements. She explored bodily identity phenomenologically and in the context of medical interventions, and analysed bodily adjustments, bodily technology, plastic surgery and so forth. On this basis, Slatman proposed that we can adjust to dramatic bodily changes and tolerate strangeness (the bodily aspects that we cannot directly experience or fully know) precisely because of this incorporated strangeness. This results in “a paradoxical idea of identity: that which makes me `I´ is something that is simultaneously own and strange to my `me-ness´ (p. 20). The strangeness relates to the substance-dimensions of the body, to which we have a certain distance. After all, changes and processes in cells are not directly available or recognizable in our experience.

The body that we have is the body that we are, and in the moment something new occurs, like a painful injury, our relationship to own body alters. This means that the dynamics between habitual and spontaneous aspects of the lived body are central to bodily identity and are foregrounded when living through bodily changes. As we coexist and interact with others in a shared world, other people’s gaze and possible judgements or rejections raise our awareness of how the body visible to others, is the body that we have. Hence, *body image* affects how we experience ourselves. Bodies vary and change, and human beings grow and alter as life unfolds. Although the body changes both invisibly and visibly in illness, through medical interventions and aging, we continue to experience ourselves as the same person. We do not become someone else (Slatman 2014). New experiences, perceptions, practices and emotions keep coming and replacing those that we once had, yet some of our experiential domain holds on to a certain continuity.

Ricoeur (1994) elaborates on identity in the tension between innovation and sedimentation. He proposes that a person’s identity emerges in the identity of this person’s story. Identity can only develop through communication. It is in the construction of a story about life experiences that identity comes into being when the acting person transfers events into plots. Thus, identity is structured in the structure of the narrative. This means synthesizing a complexity of events and actions, and configuring these in relation to what passed and endure. Narrative identity thus generates from the interpretation of action in time and is shaped in the dynamic between two overlapping poles of the self. The storytelling pole expresses selfhood as consistency (*ipse*).^1^ Over time this consistency becomes sedimented as characteristic traits of a person’s self like habits, roles etc., which can be recognized as the same over time (*idem*).^2^ Thus, *what* we are (idem/sameness) and *who* we are (ipse/selfhood) overlaps. Selfhood depends on the support of sameness, without this support identity may be impaired by lack of stability. On the other hand, if sameness overshadows selfhood, a rigid personality may portray dominating character traits without the capacity to adjust to the varying challenges during life. Identity is not static. Driven by the tension between life as lived and life as told vast variations are possible in the configuring process (Ricoeur 1994).

Creating a coherent life story involves one’s ability to link diverging events and actions and to seek a sense of coherence in phenomena like memories, goals, habits and values, reinterpretation, and creative imagination. The spiral force creating identity depends on our imaginative capacity while moving from life to story and from story to life. When telling someone about an experience, understanding of meaning arises from how the storyteller structures the story and forms themself through narration. Stories may give coherence to the drama and the messiness of life in temporal order with beginnings, middles and ends, yet there are times when “the past bleeds into the present” and tends to interrupt order and plot (Riessman 2015). Narrative approaches hold potential to account for unexpected, unintended and complex turns in life stories, and also to indicate what is not straightforwardly narrated. They do not rely entirely on the storytellers’ capacity, they also depend on what narratives can be told in a specific context (Tengelyi 2018). Narratives told about the self may not be as orderly as they are often taken to be, and the self in action and experience may not be as messy (Mattingly 1998).

Narrative theory acknowledges that there is a tension between life as lived and life as told (Kristensson 1994), but there is a criticism that a narrative approach to selfhood tends to reduce bodily being to narration and language, and aim for too massive and ordered a representation of life stories (Tengelyi 2018). However, bodily being not only arises from the lived body, it also holds what we might become and our narration of it. According to Ricoeur, human life is deeply connected to the lifeworld. One’s own body is the mediating structure of being in the world, and the earth is the “mythical name of our corporeal anchoring in the world” (Ricoeur 1994, p 150). Here, identity is about body, experiences and world united. The body is a dimension of oneself, and the imaginative variations around the body are variations on the self.

This existential underpinning of bodily being in the world appears to follow the same line of thought as Merleau-Ponty who connected sedimentation to embodied experience, and its potential power to effect, restrict or release our bodily becoming (1945/2012). Merleau-Ponty pointed to repeated practices, attitudes, norms and beliefs that can acquire preference and become habituated and incorporated, and which are not easy to change (Zeiler 2013). We are and become ourselves in tensions between what stays the same and what changes, between being ourselves and becoming ourselves in encounters with others. According to Ricoeur, acquired identification incorporates *the other* into the composition of identity in the pole of sameness. We recognize ourselves in other people and cultural heroes, and as we develop loyalty and fidelity to them their norms and values become internalized in our character traits (selfhood).

Essential to narratives is that one thing happens in consequence of another. This is commonly referred to as narrative causality. Storied description is a human way to find coherence and meaning. To what extent narrative expresses the shape versus its meaning is debated. The core question is what is primary, experience or narratives? A humanistic, person-centred approach emphasizes lived experience primary to narratives. Narratives express action and experiences in individual meaning making. Agency and identity evolve through structured stories representing the individual (Squire et al. 2013). Structuralist and poststructuralist critiques are that cultural narratives exist before action. Cultural script guides human conduct and meaning making and events imitate stories already woven into the sociocultural world. Culturally shaped narratives act as prototypes or templates and serve as scripts shaping action and meaning making. Experience is thus an enactment of pre-given stories. This means that stories act to make life social, but people may always choose the stories they grow up on (Frank 2010, p. 24).

Mattingly attempts (2010) to bridge the structural- and the experiential by integrating narrative and phenomenology. She develops narrative phenomenology as a theoretical lens to open an existential window for particular people to reveal something about the struggles of the many (Mattingly 2010, p. 8). By this we understand that a specific narrative unfolding in a given context with unique individuals may express something universal which is relevant and recognizable to other people. The narrative is then expressed and reshaped by the person’s unique point of departure and specific context. Both Frank (1995, 2012) and Mattingly (2010) emphasize the role of narrative resources and the culturally shaped core narratives. Such narrative types are trajectories for people to tell their own unique stories. Within the dramas of health care, illness and healing canonized medical genres like in detective, battle and repair stories can be identified (Mattingly 2010). Illness narratives of the suffering may be narratives of restitution or chaos (Frank 1995). Healing as a transformative journey proposed by Mattingly and quest narratives as proposed by Frank are narrative genres displaying how people change and deal with suffering and challenges over time.

Culturally shaped narratives of medicine and health care influence expectations and experiences of illness and health. Moreover, expectations of positive health outcomes and socially rewarding changes following WLS tend to take precedence over stories on fluctuating weight, problematic eating and illness: “Weight regain stories are easily silenced, buried beneath social, cultural, and institutional/ medical narratives of successful weight loss and transformation” (Groven and Glenn 2016).

### Weight loss for health and wellbeing: cultural narratives at play

In western societies, there is a constant push towards lean, active and productive bodies, supported by health authorities, popular culture and governments (Lupton 2012a, 2012b). *Healthism* indicates a particular way to situate health problems and their solutions at the individual level, shaping popular beliefs on illness and health (Crawford 1980). On this notion, health is a subjective matter, the premise for our well-being, and a goal we can reach primarily through modification of lifestyles, either self-directed or with the help of health practitioners or therapists. Being larger bodied does not align with cultural norms and how people should present and live, and is a vulnerable situation saturated with moral meanings.

Weight issues are primarily faced as self-inflicted, a sign of personal failure, potentially shameful, triggering blame or pity (Puhl and Heuer 2009; Puhl et al. 2013). Living as large and being an active participant in society is hard, as *weight stigma*, discrimination and feelings of body shame and guilt are prevalent and alienating (Ramos Salas et al. 2019). Weight stigma is at play in social interaction, for example, when someone assume that they know something about another individual and her life because of her bodily appearance, and therefore has less interest in getting to know her, hesitates to accept her, discriminates or harasses her. In contrast, practices of self-improvement and disciplining the body attract positive recognition. Engaging with diets, exercise and other weight loss practices signals personal strength, mastery, productivity and independence (Bordo 2003).

The *obesity epidemic* or *pandemic* are popular concepts to illustrate the rising prevalence of overweightness and obesity in several countries as an alarming and deeply concerning trend (Ng et al. 2014). Hence, monitoring populations’ weight and developing strategies for obesity prevention and treatment have become important health priorities.^3^ From a *medical* perspective, high level of body fat equals health risk and indicates increased sickness and costs. This means that living as large attracts both medical and political attention, as health resources are scarce and costs related to obesity are significant (Tremmel et al. 2017). Consequently, the prevailing narrative is that of excess weight as dangerous, unwanted and unsustainable.

*Severe obesity* involves a health risk and potentially suffering and social burdens.^4^ Therefore, health authorities emphasize obesity prevention and offer help for severe obesity, in some countries even financed by the state. Medical interventions for severe obesity are typically lifestyle programs, medicine (pharmacotherapy) or surgery. Surgery is popular worldwide because of its effectiveness compared with lifestyle approaches and medical treatment, resulting in larger and more sustainable weight losses and health benefits (Adams et al. 2007; Karlsson et al. 2007; Arterburn et al. 2015; Schauer et al. 2017). However, the treatment is invasive and patients risk unintended consequences, as the long-term effects are not clear (Colquitt et al. 2014; Puzziferri et al. 2014; Courcoulas et al. 2014).

*Weight regain* is a common phenomenon after weight loss, including after WLS (Velapati et al. 2018; Santos et al. 2017). Most nonsurgical weight loss attempts fail within the first year and regain is prevalent (Wing and Phelan 2005; Dombrowski et al. 2014; Thomas et al. 2014). Patients with weight regain after WLS have reported higher rates of problematic eating/eating psychopathology afterwards (Mauro et al. 2019). Despite the risk of weight regain or side effects following surgery, for people of size, surgical treatment remains an opportunity to change their bodies and lives via weight loss, offering more optimistic future prospects on health and longevity. In this, WLS facilitates a *transformation narrative* (Bocchieri et al. 2002). However, ambivalence, risks and uncertainties about the future leave this narrative open ended.

Furthermore, medical and experiential narratives seem partly incongruent, favouring normalization of bodies and lives over uncertainties, failure and vulnerability. Severe obesity is thus situated in a tension between canonized medical narratives where surgery appears to have much to offer, and other cultural narratives of living large as de-valued and shameful, partly related to the medical narrative. Stuck in between are the personal narratives of hope, joy, disappointment, and the fear of failing to transform. In the becoming of, being and combating a large body, this tension seems like a kernel as a driving force throughout a lifespan in persons’ lived experiences after WLS.

## Methodology

Narratives are at the core of human life and have certain capacities: “Stories give form—temporal and spatial orientation- coherence, meaning intention and especially boundaries—to lives that inherently lack form*”* (Frank 2010, p. 2). We live through and embody our experiences, but do not fully understand them, for example when undergoing major bodily changes. Narrating our story gives the opportunity to form and understand life experiences, by revisiting the past, holding on to the present and orienting to the future (Frank 2012).

Narrative research is multidisciplinary (Squire et al. 2013). The current research is a case study theoretically based in phenomenology and narrative theory. *Narrative phenomenology* bridges discrepancies rather than polarizing them, and offers a position from which to see how lived meanings, desires and fears saturate the past and present (Mattingly 1998, 2010). Narratives that are embodied and aesthetically based are significantly phenomenological*.* Still, they are not a naïve mirroring of reality but emerge from the lifeworld as interpretations. This methodological approach “allows us to give an account of historically particular social interactions and even personal experiences, while situating these extremely situated events and experiences within larger political and social frameworks …” (Mattingly 2010, p. 217).

Narrative phenomenology endeavours to acknowledge narratives as scripts, but also the impact of immediate context and, to recognize the role of the discourse on shaping meaning making, but also to pay attention to the non-linguistic/non-verbal/sensuous influence. It does not just account for meanings shared by cultural groups, but also contribute with tools for interpreting personal meaning, the inner landscape of an individual's motives, desires and perceptions (Mattingly 1998, p. 44).

From the perspective of narrative phenomenology, everyday experiences are valuable sources for philosophical exploration as well as a rigorous basis for scientific knowledge. To acquire the meanings of a story told, the listener must allow themself to be drawn into the story, to be moved by it and to let themself be affected by it in an existential way, according to Ricoeur. Frank underscores that the listener must be particularly aware not to impose other stories on the narrator’s own. This involves allowing oneself to be moved by the impressions of Thea’s story, but it also requires sensitivity to medical- and popular narratives on health, weight and lifestyle, and how those culturally shaped narratives act and effect people and cultures. The narrator speaks with one voice, yet the voices of significant others merge into that voice. Illness narratives typically are *polyphonic*: a mix of one’s experiences, thoughts and feelings, medical professionals’ information and expectations, loved ones’ care and worries and fellow ill’s hopes (Frank 2012). Adding to this complexity is how this research material is developed in co-construction between interviewee and the interviewer, in the context, time and place of the situation in which this research material developed.

### Narrative interviews and the research material

In narrative interviews, the aim is to capture stories about particular events and develop a full story (Pederson 2013; Riessman 2008). In this study, we explore Thea’s life history, her life trajectory, her experience of undergoing WLS and the mutual connection between life story and battling with weight. We utilized open-ended interview questions about childhood, adolescence and family, weight, food, eating, health, illness, activity, rest, and relationships. Thea, the protagonist in this story, and the first author have corresponded by email and met for two interviews. The purpose of the first interview was to acquire her life story. We did the second interview after initial analysis, to elaborate on meaningful events and certain aspects of the full story (see analysis). We prepared new questions for interview two, which was longer and included lunch to create a good atmosphere for reflection. For both interviews, we met in a quiet room at a hotel nearby where Thea lives.

The recorded interviews lasted for two hours and five minutes and three hours and forty-five minutes, respectively. Directly after the interviews, the first author (EN) wrote a reflective note on the immediate impressions from the dialogue. A research assistant transcribed the interviews verbatim. The empirical material consists of email correspondence, reflective notes, recorded interviews and transcripts.

### Analysis

In narrative analysis, it is not the parts of the interview materials that are significant, but how the parts connect to build a meaningful whole (Josselson 2011). This means that the narrative analysis is a hermeneutic process: We read the parts in light of the full story and vice versa. Continuity, change and bodily experience across many years were central in the current material, and we analysed the material as an account of which different passages were in dialogue with each other, the past and the present. Linking and ordering events required interpretation. When building up the coherent narrative we searched for links between different events and phenomena in order to bring possible narrative causality to the fore. Narratives carry the structure of a beginning, middle and end, evolve in a particular setting and always relate to values and temporality. A step-by-step approach to narrative analysis does not exist, nor is required for a sound analysis (Frank, 2010). Rather, narrative research gives priority to approaches that are fruitful and productive in the process of capturing lived experience, meaning making and insightful analyses (Josselson 2011).

Initially, EN last author (RS) read the material separately to get an overview and a sense of the structure and central themes and narrative threads. EN listened to the interview while rereading the whole transcript slowly, line by line. Weight and body struggle appeared as a winding path meandering around themes, for example “being me in my family”, “becoming a patient with severe obesity” and “becoming who I am”. We discussed initial themes and EN reread the material, searching for contradictions, emotion-laden events, actions and insights connected to the life story, the lived body, weight loss, and expressions of selfhood. She reorganized the material according to time and the central topics of own body/weight, food/eating and self/other, and wrote a coherent story.

Thea expressed that she wanted to be included in the process and took initiative to read and comment on the coherent story. She responded to the story in emails, filled in some details and commented on certain aspects. For example, she commented that reading about her childhood was somewhat strange, that it was striking and emotional. Moreover, she expressed that her situation regarding food had not changed during the years we had been in touch, whereas her weight still fluctuated and was demanding to control. For further elaboration and deepening some of these aspects, events and passages in the coherent story, we agreed to meet for a second interview.

We read the materials again with attention to possible multiple voices, and three plotlines came to the fore (1) *hunger* (2) *success/transformation* and (3) *fighting weight*. EN started writing on the basis of these plotlines and the empirical material. In the process of writing, discussing, re-reading and re-writing, we identified a web of varied narrative threads including striking contradictions.^5^ Interwoven in her life story, WLS stand out as an essential turning point. We have shaped and reshaped the plots during the process of writing.

Below, we present our analysis of Thea’s story of her life and her body in two distinct narratives. By disentangling Thea’s story into two separate storylines we seek to illuminate paradoxes within her long-term weight loss processes. In Thea’s life, however, these two storylines are intimately entwined. The first is from a medical perspective: *Thea’s Medical Weight Narrative: From Severely Obese Child to Healthy Adult*. This is followed by theoretical reflection on what kind of transformation this narrative holds. The second is *Thea’s Story: The Narrative of Fighting Weight*. This narrative unfolds Thea’s transformation as a journey and her ever present quest to solve her weight problems*.* Different from the medical narrative, the second narrative has strong connotations to two core narratives suggested by Mattingly (1998) and Frank (1995). Their two core narratives of healing as a transformative journey and as a quest both depart from the first-person perspective and have the capacities to give voice to Thea’s process of change over time. Her life-experience from early childhood to the present time are structured into four time-epochs with theoretical reflections after each.

## Thea’s medical weight narrative: from severely obese child to healthy adult

Thea weighed 36 kilos at the age of seven and 84 when she was twelve. When she was 115 kilos, at the age of fifteen, she stayed several weeks in a hospital in order to lose weight. What she lost, she quickly regained, and weight continued to escalate and reached 140 kilos at the age of twenty, when she underwent Jejunoileal Bypass.^6^ For Thea this surgery was effective. She lost 40 kilos in nine months. She had no severe side effects but suffered some stomach pain/diarrhoea after overeating and, now has early stages of osteopenia.^7^ She has undergone several plastic surgeries to remove redundant skin. She joined a commercial diet program based on diet, exercise and cognitive strategies, lost another 20 kilos and for a few years her weight was stable at about 80 kilos. Since then her weight has fluctuated between 80 and 110 kilos, and currently, she weighs 84.5 kg.

Thea is living alone. Long-lasting musculoskeletal pain led to early retirement and disability pension about the age of fifty. She still considers her weight too high and aims to stabilize at 80 kilos. She weighs herself daily, exercises regularly and systematically diets and fasts. She has some support from her general practitioner (GP) and a local obesity clinic. Overall, Thea is in good health and takes good care of herself.

WLS is considered to facilitate a transformation narrative (Bocchieri et al. 2002), however it is a surgically induced transformation with connotations to the canonized medical genre of repair. Surgery repaired the malfunction of Thea’s body regarding severe obesity. From a medical vantagepoint this is a story of success. Her body no longer requires medical intervention and even 45 years after surgery, her body mass index is within the acceptable zone. However, it is narrated from the outside, deprived of lifeworld experiences and, success seem to eclipse the endless battle she has been fighting. The repair or transformation is partial since Thea’s urge to overeat remains the same, and she is still fighting the battle, alas she is now doing this on her own.

## Thea’s story: the narrative of fighting weight

### Growing up as a large bodied girl—standing out

Thea grew up in a family of five, living rurally on the coast of Norway. Her mother was a housewife, but her father worked on a ship, and was rarely at home. From early childhood, Thea had noticed she ate more than others. At the age of three she used to eat all the leftovers. She coined it *being born a food-wreck*. She recalled herself a large bodied girl with a ravenous appetite and a desire to eat more and more.

When starting school, she saw herself larger and heavier than other children, and her body grew fast. She had friends and did well in class, but from the first day her weight became a problem. Exclusion and bullying became part of her daily life, but Thea had fought back: “I was tough, a real hard-ass. I developed a ‘thick skin’, and as I was bigger and stronger, I beat them up.” Not everyone bullied her, but no one stood up for her. Moreover, her body kept getting in the way when playing with friends. She could not run as fast and climb or jump as high as they did. She could not get into exciting narrow spaces, where her friends could easily crawl in and hide. She often found herself alone and angry, and sometimes she got into trouble. Once, her friends had climbed onboard a large fishing boat. She got so angry when she could not get herself onboard, that she loosened the boat’s mooring. Luckily, someone saw what happened, and saved the situation.

Despite challenges, Thea emphasized that her childhood had been good. She endured and mastered her body as well as she could. However, her body-size came between herself and other people and this had been an issue in the family. When her mother had tried to help by controlling her eating, it just made Thea angry because she was denied what her sister got. Her sister had breakfast in bed, because she was so thin, but Thea was too overweight to deserve this. If her sister did not like what they had, something else would be prepared for her. This was never done for Thea. She never doubted her mother’s love, but it felt deeply unfair to be treated differently because of her body and to be punished for something she could not help. This had created a distance to her mother and sister and made her feel inferior.

Regulating food triggered conflicts and shaped her role in the family. Thea became jealous of her siblings, especially the youngest one who was the “miracle and the extraordinary child”. Thea vividly remembered her first day at school. Her sister had to take her because the mother had just given birth to number three. Her sister had not wanted to, because Thea was so thick. For the same reason she did not let her be her bridesmaid when she got married. Thea can never forget this: “I have told her many times what she did to me.”

Thea had never explained to her mother how sorry she was for the inconvenience her weight had caused, such as finding clothes that fitted and getting properly dressed. She remembered how her mother had to take her to the men’s department where brown plaid pants laid out nicely organized according to their waistline. For her confirmation ceremony, Thea had to pay a woman to sew a dress, but after that Thea started sewing her own clothes. As Thea kept growing, her mother searched for help. The GP had thought she would grow out of the *puppy fat*, but when she turned fifteen, he referred her to a hospital for dieting. She lost some weight, but quickly regained all of it and more. From this point, Thea’s story conveys her body as a severe problem, requiring intervention and medical attention.

Thea’s appetite comes across as an innate trait of what she is, whereas the awareness of her body size emerges in interaction with the physical and social world. Either by physically getting stuck and comparing herself with other children, or eating practices shaping her role in the family, involving favoritism, control and jealousy. In light of Ricoeur’s notion of narrative identity Thea’s appetite is persistently the same (idem) throughout this period, but her awareness of being oversized emerges in stories of who she is becoming in interactions (ipse), such as not wanted as bridesmaid. Interaction with physical space and intersubjectivity shaped the vision of herself as being bigger than others. Over time, these experiences seem to start sedimenting into her body image, becoming a large-bodied person as a character trait (idem). As Ricoeur underpins, selfhood (ipse) and sameness (idem) overlap in the dynamic development of a person’s identity where building new stories on significant life events becomes overshadowed by sedimented traits. This shift is also visible in the medical perspective where the understanding of Thea’s size shifts from innocent puppy fat, which she will grow out of (change with time), to becoming a condition of medical concern. Who Thea is (ipse) also unfolds in narratives of fighting back, resistance to attempts to control her eating, and learning to sew because clothes are hard to find. Anger, jealousy, loneliness, exclusion and bullying. Even so, she feels loved and experiences her childhood as good. Her selfhood portrays a strong will to hold her own.

### Leaving home—loneliness, overeating and gaining weight

Despite her struggles, Thea had a strong sense of belonging, beeing rooted in her family, friends and the small community: “I felt so safe there, I felt protected. Everybody knew of my body and me.” Moving to a new town before she was 16 to go to high school was hard. She was not ready to leave her family and manage on her own. Connecting with new people was difficult. She recalled:

“Everything got worse. On the first day, no one spoke to me. I noticed a few gazing at me, then smiling to each other and I immediately knew that my appearance amused them. During a class break, while I sat on a desk, two girls put a mark on each of my sides. When I got up, they measured the width of my buttocks.”

Being a large-bodied newcomer at school and in a new town was difficult. Harassment, self-contempt, and no longer being watched by her mother eating had accelerated her difficulties. Homesick and lonely, confused and bewildered, she ate to relieve her pain, but this just made it worse. This vicious circle made her feel hopeless and ashamed. She gained 24 kilos the first semester, and she still remembers the shock on her father’s face when she came home.

Making friends with another large-bodied girl had helped, but she still struggled, particularly in relation to men. Late at night drunk men who had not found anyone would come to her, expecting her to be happy that someone wanted to take her home. She sighs: “All these painful experiences have made me who I am. So many situations hurt me, and that I cannot forget. Lots of alcohol and everything happening in the dark.” She felt shameful and gained more weight. Daily life motion became increasingly strenuous. However, Thea was eager to learn, so she moved to a bigger city for further education. With a good result she soon got her first employment. Everyone had stared at her when she first appeared in the office. Still, she felt okay and became increasingly confident. The manager acknowledged her skills, but job satisfaction did not prevent her from overeating.

Thea’s problems peaked in a critical phase of life, namely adolescence.^8^ Developing personal autonomy, becoming less dependent on her parents and more dependent on friends involves complex processes and contradictions. Relations to peers involve friendships, joy, excitement and security, but also comparison, competition, exclusion and ruptured relations (Erikson 1968). Being accepted by friends and avoiding social rejection is important for adolescents’ sense of social- and personal worth (Blakemore and Mills 2014). Developing a sense of self, realizing one’s own potential and creating future capabilities in continuous interaction with the social environment, while at the same time being extremely sensitive to the social environment is challenging (Patton et al. 2016). Leaving home at sixteen, Thea was still someone’s daughter, sister and friend, but she approached the world as an individual. She had experienced social exclusion, rejection and bullying directed to her appearance and body size, and ended up being mostly alone, ashamed of herself.

With Thea’s claim that the painful experiences have made her who she is, Thea underpins how identity emerges from life experience. The vulnerability of human intersubjectivity becomes evident in her adolescent life experience. New stories unfold and the sedimentation of her as large bodied is reinforced. Ricoeur underscores that selfhood needs support from sameness. Without the stabilizing sense of safe belonging, where she was recognized as the same, her selfhood is missing important support dealing with new challenges. In the new context Thea’s sameness does not seem to support her selfhood, especially in the accounts of events associated with devaluation and shame. Nevertheless, her agency and capacity to hold her own hold points to a different future, despite her appetite and hunger for food remaining the same.

### Having surgery for weight loss—restart, reconnection and relationships

When she reached 140 kilos “just existing was too heavy” and by chance a physician had suggested surgery because her bodyweight was considered a threat to her health. The physicians had been clear. No-one knew how surgery would turn out in the long term. For Thea it was obvious: “I had no choice.” It was a turning point. Thea underscored that this physician had saved her life. She travelled alone to Oslo. The surgery took a long time. She remembered waking up from the anaesthetic and having her bowels work again. Everything that came out was weighed. The surgeons had worried that the wound would not heal properly due to all the fat, and it had taken time. “Most of all,” she said, “I remember the flight home, because I was not well at the time. I went home to my mum.” She needed to be cared for.

Finally losing weight was amazing: “How easy it was to walk! Just buy a bike and take up cycling. It was fantastic!” The only minus was diarrhoea. She ate like she used to, and still lost weight, in the beginning. However, her weight loss plateaued, and when she started regaining, she realized she needed to do more. This is when she joined a weight loss and lifestyle program. The surgically induced weight loss initiated this new way of dealing with her weight problems: “I suddenly felt in control and got a grip on myself”.

Thea described herself as “not that into other people”. After weight loss she was still vulnerable regarding her body, but stronger in herself. She engaged more in building relationships, reconnected with her family, a few close friends and her relationship with men altered. In her forties, she fell in love with a man she had known for years. She said: “With him, it was different. We have never hidden in the dark or needed alcohol to connect.” With him she felt good enough and loved for who she was. She treasured their mutual respect and long friendship, companionship and love, and was deeply grateful for it. Nobody else had seen her naked body. With him, Thea was comfortable in her own skin, and she had never been ashamed of her body. They did not live together, but were cuddly, affectionate, intimately and bodily connected. She cared about him and trusted the love he showed for her.

Surgery is the turning point in Thea’s life story, the onset of a new beginning. Her body is changing. How does this change her narrative identity? With surgically induced weight loss her body is not the same. What she *is*, as her characteristic large body, is changing. We assume that this may influence the dynamic of ipse and idem, and that the body as her own became more supportive of who she wished to be. This in turn is more congruent with the appraised cultural narrative of self-improvement through diets and exercise (Bordo 2003). Identifying with cultural norms which favour personal strength, mastery, productivity and independence might have become more achievable for Thea. Losing weight also made movement and exploring her body in new ways more possible and enjoyable. According to Ricoeur this would integrate in her pole of sameness. From this turning point new stories come into Thea’s selfhood. She describes a sense of getting a new grip on herself, indicating a new sense of agency with a new kind of control supporting her discipline with regards to eating and exercising. Narrative threads of new social experiences add to the unfolding of her selfhood. Why then does Thea still consider her body weight problematic?

### Living with the scars—fighting own body

After years of weight loss, surgeries and exercise Thea’s body is mostly toned and firm but she explained how the scar tissue has affected self-perception: “My body looks terrible. It is awful, looking like a Frankenstein monster. It looks as if it is pieced randomly together of different stuff and without symmetry.” Exercise in water would be good for her musculoskeletal pain, but to show herself in the swimming pool is unthinkable. After surgery, her body shame also included her skin. Despite weight loss and change, she remains extremely vulnerable regarding her own body. She remembered the sting when coming into the office where two of her best friends sat grinning: “I was sure they laughed about me. I could not go to them. I turned and went back outside.” This was after she had lost weight she explains: “So I was no longer large, yet I still was.”

In many ways Thea is satisfied. Traces from her time as being overweight are there, but they do not interfere with her relationships with important people in her life. Being able to help her family means a lot to Thea. For years, she and her older sister lost touch, but this relationship is restored. Severe illness has made her sister need Thea’s help. Thea dreads the thought of becoming dependent on others, she plans to stay healthy and manage alone for the rest of her life. Losing the magical five kilograms is what she needs to achieve a good life, and she is nearly there:

“My economy is great, no fuss there at all. I have taken firm control of everything (short pause). Yet not of food and eating. It is so strange. I am in such control of my life, and then everything goes up in flames regarding food. I just cannot get it. Why can I not just have a little? Why do I need so much?”

Daily exercise, controlling food and weight is her lifestyle. The passion for food keeps undermining her project, leaving her trapped in the pendulum of overeating and dieting. She allows herself unrestricted eating one day while dieting the rest of the week. To stop is hard and she often slips into overeating for several days: “I keep falling off the wagon.”

Without dieting Thea gains weight. She described a race with no finishing line: “It is like a hamster wheel.” She is exhausted from the intense and never ending efforts of wrestling to control her weight and eating. She wants to change, yet fears it. To abandon the idea of losing more weight involves a deep sense of insecurity. She yearns to find peace with her body, but she does not know how to. Thea is an expert in self-improvement practices, and to let go is risky. To settle with her present weight means increasing little by little. “I must return to 80 kilos. Then I can relax again,” she said.

Thea has fought weight for a long time. She does not see herself as strong compared to the large bodied little girl she used to be. She is more vulnerable now. Since surgery, losing and keeping off weight has been excruciatingly hard work. Her dilemma is how to end the weight loss process and find peace with her body, without relapsing:

“Food and weight have controlled my life to the present day. Today I have control, but it has not always been like that. I have fought against the kilograms all these years. Every day is a struggle. Now, I have had a bad period and gained a few kilograms, but I will turn it around.

Remaining vulnerable about her body indicates that the sedimentation of the body image as large bodied overrules and eclipses Thea’s new experience of becoming normal sized. According to Ricoeur (1999) narrative connectors help the configuration of events into coherent stories by bridging experienced time and objective time in what he calls narrative time. This is essentially what human time is. As narrative connectors, traces exist in present time due to a no longer existing past. Thea is no longer overly large, but traces of the past remain in the present time, in her body image within her narrative identity. The scars do not let her forget. Slatman (2014) proposes that we can adjust to dramatic bodily changes and Thea’s body has changed dramatically. She is pleased with the reduced weight. However, her use of the Frankenstein metaphor suggests that there is a strangeness which is hard to adjust to. This creates a sense of distance to her body; the metaphor may act as protection against potentially devaluating gazes.

According to Dolezal (2015), body shame and life experience mutually shape each other. Weight surveillance tends to inflict body shame and self-consciousness. Moreover, turning one’s attention inwards, towards oneself, can interrupt meaningful engagement with the social world, activities and life projects. Thea’s preoccupation with weight control does not seem to inhibit her social engagement. On the contrary, responsibility and care for others are at her core and are deeply meaningful to her. When her close friend cares for her, she rests. With him, she is free of body shame. Hence, the current analysis suggests narrative plotlines where bodily being, social rejection and belonging as well as problematic overeating entwine. This illustrates Ricoeur’s emphasis on narratives as a synthesis of the heterogenic.

Two forces seem to drive this plot. The desire to eat remains the same throughout. The capacity to hold her own changes in the duration of time; in early childhood and adolescence this capacity seems to oppose or undermine attempts to change the body from increasing. After surgery, however, this capacity turns into a strong will to overcome the weight problem, but surgery did not influence the drive within her to overeat. It puzzles Thea why she cannot control this, as if there is something in her body which is out of her reach, something strange or other than herself. The desire to overeat keeps undermining Thea’s deliberate will to overcome it. Ricoeur’s notion on the *wounded cogito* reminds us that there is always something which is strange within us, and that self-reflection depends on interpretation and communication.^9^ Thea’s narrative resources do not suffice in her attempts to grasp this side of herself. One might say that her *cogito* does not control this desire. She appears to be trapped in a conflict between these two opposite forces within what she calls the “hamster wheel.”

### Final reflection

In this case study Thea’s lived body is the pivot. We have followed her journey from becoming an obese child to her present life. The main turning point of the story is when her weight had reached 140 kilos and WLS sparked hope for a new life. Surgery was lifesaving according to Thea. However, the surgical transformation did not suffice. It did not influence her desire for food. In the narrative genre of transforming the body by repair, her relationship with food did not fit into the plot. Gradually, Thea identified overeating as fundamental to her health and wellbeing and tried to find ways to make her peace with eating. According to her, neither her GP nor the health practitioners at an obesity clinic could offer appropriate support. Encountered as WLS-patient she is still met merely through the lens of obesity as health risk. What is on offer is lifestyle advice only. Her problematic eating practices remains unaddressed.

As “nearly normal weight” she presently lives an independent and satisfactory life, but the never-ending weight cycle has worn her out. Her story highlights that WLS can involve long-standing ambivalence and distress that is hard to resolve alone. To keep fighting her own body seems less complicated than making peace with herself. To address what drives her overeating might be worth trying, rather than more self-discipline. Thinking with Ricoeur, this calls for communication with somebody who will give space to investigate possible interpretations as a means to resolve this consuming conflict. This help she has requested has not yet been offered. Even so, Thea has hopes for the future, if only she could find her way out of “the hamster wheel.” Her quest is still how to deal with overeating which seems to be the prerequisite to a good life.

## Methodological consiserations and thea’s participation

As physiotherapists and health researchers, reflecting on our preunderstandings was important in order to give space for Thea’s story to unfold. This implies fluctuation between becoming aware of what is “ours”, holding back and actively using it to form analytic questions and by drawing on theory. For example, our knowledge about phenomenology, narratives, the lived body and long-term experiences of WLS was advantageous while planning, preparing for data production and writing. Nevertheless, this required reflection particularly during analyses.

Capturing and investigating a life story on the initiative from the protagonist herself, was a new and interesting approach to research for us. Ethics and validity related to how the study begun and developed, for example, when Thea explicitly wished to follow the research process, stay in touch, read and comment. We welcomed that and adjusted the research design accordingly. For example, her responses to our initial analysis actualized a second interview, which became more of a dialogue and probing for the complexity of Thea’s story. We focused on emotional events and open reflection from both parties was essential. This allowed for Thea to elicit her experiences and recollections of episodes and situations, and to find her words and unfold her story. We were conscious that our investigation was a part of Thea’s own process of trying to understand and make meaning of her experiences. It was a fine line between interviewing for research, probing for concrete details, listening to the stories, and being supportive. As an experienced research interviewer and physical therapist, the interviewer sensitively related to the vulnerability of the situation and her responsibility to respect that. By involving herself in the research process Thea introduced a participatory research approach into the study. Her response after reading the analysis of the first interview was that it made her capture and formulate what was at stake in her story:

“It is somewhat emotional. That girl was I (with emphasis). I was just a little girl. Having problems with my body as a child left an imprint on me, because I felt that I was of extremely little value back then, when I was thick. Totally worthless. Being physically unable to do what other children did was difficult. I always fell behind. Particularly the food-part strikes me, touches me. That (eating) is at the core, it is everything, really*.”*

## Footnotes

1 Ipse is from latin and means «self».

2 Idem is from latin and means «the same».

3 To monitor weight in populations and individuals across insurance, medicine and public health worldwide, the Body Mass Index (BMI) is a popular measure. BMI is a measurement for the ratio between body weight and height in adults, defined as weight in kilograms divided by the square of the height in meters/ kg/m^2^. The higher the weight, the higher the risk.

4 Severe obesity is a medical term indicating a considerable amount of excess body fat and high weight, defined as having a Body Mass Index (BMI) of ≥ 40, or of 35 with weight related comorbidities (cardiovascular diseases or diabetes type 2) (World Health Organization 2017).

5 Threads: Daughter, sister and family issue. Friends, play and bullying. Mother’s worries, care and punishment. Belonging and isolation. Missing home and gaining weight. Harassment, self-loathing and losing control over weight and eating. Withdrawing socially and thriving in education and employment. Rock bottom and life changing weight loss. Social reconnection, love and longing. Thick skinned and uncomfortable in own skin. Full control and letting go. Exhaustion and motivation. Trusting own body and dreading weight gain. Finale and failure. Reaching the finishing line and being ambivalent about crossing it.

6 Jejunoileal Bypass was a widely used surgical procedure during the 1970s (Moshiri et al. 2013), but was abandoned due to several severe complications (Welsh and Murayama 2018). Reversals or revisions have been necessary for a majority of patients (Moshiri et al. 2013).

7 Osteopenia means that a person has weak bones and not yet osteoporosis.

8 Adolescence is the period starting in puberty (biological and physiological changes) and ending with various forms of social adjustment, with relative self-sufficiency or reaching towards a stable adult role in society (Blakemore and Mills 2014).

9 In his early work Ricoeur explored the voluntary and the involuntary in human agency, underpinning how rational understanding is limited by the embodied and situational (Ricoeur et al. 2002). He described that immediate self-experience is impossible; we do not have full access to ourselves. Self-reflection depends on interpretation and communication. Hence, *cogito is wounded* as a foundation of all knowledge.

## References

[CR1] Adams Ted D, Gress Richard E, Smith Sherman C, Chad Halverson R, Simper Steven C, Rosamond Wayne D, Lamonte Michael J, Stroup Antoinette M, Hunt Steven C (2007). Long-term mortality after gastric bypass surgery. New England Journal of Medicine.

[CR2] Arterburn David E, Olsen Maren K, Smith Valerie A, Livingston Edward H, Van Scoyoc Lynn, William S, Yancy George Eid, Weidenbacher Hollis, Maciejewski Matthew L (2015). Association between bariatric surgery and long-term survival. JAMA: Journal of the American Medical Organization.

[CR3] Blakemore Sarah-Jayne, Mills Kathryn L (2014). Is adolescence a sensitive period for sociocultural processing?. Annual Review of Psychology.

[CR4] Bocchieri Lindsey E, Meana Marta, Fisher Barry L (2002). Perceived psychosocial outcomes of gastric bypass surgery: A qualitative study. Obesity Surgery.

[CR5] Bordo S, Bordo S (2003). Reading the slender body. Unbearable weight. Feminism, western culture, and the body.

[CR6] Colquitt Jill L, Pickett Karen, Loveman Emma, Frampton Geoff K (2014). Surgery for weight loss in adults. The Cochrane database of systematic reviews.

[CR7] Courcoulas Anita P, Yanovski Susan Z, Bonds Denise, Eggerman Thomas, Horlick Mary, Staten Myrlene A, Arterburn David E (2014). Long-term outcomes of bariatric surgery: A national institutes of health symposium. JAMA Surg.

[CR8] Crawford Robert (1980). Healthism and the medicalization of everyday life. International Journal of Health Services.

[CR9] Dolezal Luna, Dolezal Luna (2015). Body Shame and Femal Experience. The Body and Shame. Phenomenology, Feinism and the Socially Shaped Body.

[CR10] Dombrowski Stephan U, Knittle Keegan, Avenell Alison, Araújo-Soares Vera, Sniehotta Falko F (2014). Long term maintenance of weight loss with non-surgical interventions in obese adults: systematic review and meta-analyses of randomised controlled trials. BMJ : British Medical Journal.

[CR11] Erikson Erik H (1968). Barndommen og samfunnet [Childhood and Society].

[CR12] Frank Arthur W (2010). Letting stories breathe.

[CR13] Frank Arthur, Holstein JA, Gubrium JF (2012). Practicing dialogical narrative analysis. Varieties of Narrative Analysis.

[CR14] Groven Karen Synne, Glenn NM (2016). The experience of regaining weight following weight loss surgery: A narrative-phenomenological exploration. Health Care for Women International.

[CR15] Josselson Ruthellen, Wertz Frederick, Charmaz Kathy, McMullen Linda, Josselson Ruthellen, Anderson Rosemarie, McSpadden Emalinda (2011). Narrative research. Constructing, deconstructing and reconstructiong story. Five ways of doing qualitative analysis.

[CR16] Karlsson Jan, Taft Charles, Ryden Anna, Sjöstrom Lars, Sullivan Marianne (2007). Ten-year trends in health-related quality of life after surgical and conventional treatment for severe obesity: The SOS intervention study. International Journal of Obesity.

[CR17] Kristensson Bengt Uggla (1994). Kommunikation på bristningsgränsen: En studie i Paul Ricoeurs project [Communication at the breaking point: A study in Paul Ricoeut's project].

[CR18] Landes Donald A, Dolezal Luna, Petherbridge Danielle (2017). The weight of others. Body/self/other. The phenomenology of social encounters.

[CR19] Lupton Deborah (2012). Fat.

[CR20] Lupton Deborah (2012). Medicine as culture.

[CR21] Mattingly Cheryl (1998). Healing dramas and clinical plots.

[CR22] Mattingly Cheryl (2010). The paradox of hope: journeys through a clinical borderland.

[CR23] Maria Francisca, Papelbaum Marcelo, Brasil Marco Antônio Alves, Carneiro João Regis Ivar, Coutinho Elvandro Silva Freire, Coutinho Walmir, Appolinario José Carlos (2019). Is weight regain after bariatric surgery associated with psychiatric comorbidity?.

[CR24] Merleau-Ponty M (1945). Phenomenology of perception.

[CR25] Moshiri Mariam, Osman Sherif, Robinson Tracy J, Khandelwal Saburabh, Bhargava Puneet, Rohrmann Charles A (2013). Evolution of bariatric surgery: A historical perspective. American Journal of Roentgenology.

[CR26] Ng Marie, Fleming Tom, Robinson Margaret, Thomson Blake, Graetz Nicholas, Margono Christopher, Mullany Erin C (2014). Global, regional, and national prevalence of overweight and obesity in children and adults during 1980–2013: a systematic analysis for the Global Burden of Disease Study 2013. The Lancet.

[CR27] Patton George C, Sawyer Susan M, Santelli John S, Ross David A, Afifi Rima, Allen Nicholas B, Arora Monika (2016). Our future: a Lancet commission on adolescent health and wellbeing. The Lancet.

[CR28] Pederson Sarah Nebel (2013). To Be Welcome: A Call for Narrative Interviewing Methods in Illness Contexts. Qualitative Inquiry.

[CR29] Puhl Rebecca M, Heuer Chelsea A (2009). The stigma of obesity: A review and update. Obesity.

[CR30] Puhl Rebecca M, Peterson Jamie Lee, DePierre Jenny A, Luedicke Joerg (2013). Headless, Hungry, and Unhealthy: A Video Content Analysis of Obese Persons Portrayed in Online News. J Health Commun.

[CR31] Puzziferri Nancy, Roshek Thomas B, Mayo Helen G, Gallagher Ryan, Belle Steven H, Livingston Edward H (2014). Long-term follow-up after bariatric surgery: A systematic review. JAMA: Journal of the American Medical Organization.

[CR32] Ricoeur Paul (1994). Oneself as another.

[CR33] Ricoeur P, Ricoeur P (1999). Den fortalte tid [The narrated time]. Eksistens og hermeneutikk [Existence and hermeneutics].

[CR34] Ricoeur Paul, Hermansen Mads, Rendtorff Jacob Dahl (2002). En hermeneutisk brobygger : tekster [Building a hermeneutic bridge: texts].

[CR35] Riessman CK (2008). Narrative methods for the human sciences.

[CR36] Riessman Catherine Kohler (2015). Ruptures and sutures: time, audience and identity in an illness narrative. Sociology of Health and Illness.

[CR37] Salas Ramos, Ximena Mary Forhan, Caulfield Timothy, Sharma Arya M, Raine Kim D (2019). Addressing Internalized Weight Bias and Changing Damaged Social Identities for People Living With Obesity. Frontiers in Psychology.

[CR38] Santos Inês, Sniehotta Falko F, Marques Marta M, Carraça Eliana V, Teixeira Pedro J (2017). Prevalence of personal weight control attempts in adults: a systematic review and meta-analysis. Obesity Reviews.

[CR39] Schauer Philip R, Bhatt Deepak L, Kirwan John P, Wolski Kathy, Aminian Ali, Brethauer Stacy A, Navaneethan Sankar D (2017). Bariatric Surgery versus Intensive Medical Therapy for Diabetes — 5-Year Outcomes. New England Journal of Medicine.

[CR40] Slatman Jenny (2014). Our strange body. Philosophical reflections on identity and medical interventions.

[CR41] Squire Corinne, Andrews Molly, Tamboukou Maria, Andrews M, Squire C, Tamboukou M (2013). What is narrative research?. Doing narrative research.

[CR42] Tengelyi László, Zahavi D (2018). Action and selfhood: A narrative interpretation. Contemporary phenomenology.

[CR43] Thomas J Graham, Bond Dale S, Phelan Suzanne, Hill James O, Wing Rena R (2014). Weight-loss maintenance for 10 years in the National Weight Control Registry. American Journal of Preventive Medicine.

[CR44] Tremmel Maximilian, Gerdtham Ulf- G, Nilsson Peter M, Saha Sanjib (2017). Economic Burden of Obesity: A Systematic Literature Review. International Journal of Environmental Research and Public Health.

[CR45] Velapati Saketh R, Shah Meera, Kuchkuntla Aravind R, Abu-dayyeh Barham, Grothe Karen, Hurt Ryan T, Mundi Manpreet S (2018). Weight Regain After Bariatric Surgery: Prevalence, Etiology, and Treatment. Current Nutrition Reports.

[CR46] Welsh Leonard K, Murayama Kenric M, Chand Bipan (2018). History of Bariatric Surgery. Endoscopy in Obesity Management: A Comprehensive Guide.

[CR47] Wing Rena R, Phelan Suzanne (2005). Long-term weight loss maintenance. American Journal of Clinical Nutrition.

[CR48] World Health Organization. 2017. Body mass index - BMI. http://www.euro.who.int/en/health-topics/disease-prevention/nutrition/a-healthy-lifestyle/body-mass-index-bmi. Accessed 23 August 2018.

[CR49] Zeiler Kristin (2013). A phenomenology of excorporation, bodily alienation, and resistance: Rethinking sexed and racialized embodiment. Hypatia.

